# New Insights and Future Perspectives of APRIL in IgA Nephropathy

**DOI:** 10.3390/ijms251910340

**Published:** 2024-09-26

**Authors:** Masahiro Muto, Hitoshi Suzuki, Yusuke Suzuki

**Affiliations:** 1 Department of Nephrology, Juntendo University Urayasu Hospital, Chiba 279-0021, Japan; mmutou@juntendo.ac.jp (M.M.); shitoshi@juntendo.ac.jp (H.S.); 2 Department of Nephrology, Juntendo University Faculty of Medicine, Tokyo 113-8421, Japan

**Keywords:** IgA nephropathy, a proliferation-inducing ligand (APRIL), B cell-activating factor (BAFF), galactose-deficient IgA1 (Gd-IgA1)

## Abstract

IgA nephropathy (IgAN) is characterized by immune-mediated glomerulonephritis, with the accumulation of galactose-deficient IgA1 (Gd-IgA1) in the glomeruli and increased levels of circulating Gd-IgA1 and Gd-IgA1-containing immune complexes. An incomplete understanding of the underlying mechanisms and differences in clinical and pathological features between individuals and ethnicities has contributed to the lack of established treatments for IgAN. A tumor necrosis factor (TNF) family member, a proliferation-inducing ligand (APRIL), is a crucial cytokine essential for the generation and survival of plasma cells. Recent studies demonstrated that APRIL is a pivotal mediator in the production of Gd-IgA1 in IgAN. As our understanding of the autoimmune pathogenesis underlying IgAN has improved, various pharmacological therapeutic targets, including APRIL antagonists, have emerged. Preliminary results showed that APRIL-targeting agents effectively reduced proteinuria and Gd-IgA1 levels without significantly increasing adverse events, indicating their potential as novel therapeutic agents for IgAN. In the present review, we discuss the current understanding of the role of APRIL in the pathogenesis of IgAN and novel therapeutic strategies focusing on APRIL-targeting agents for IgAN. APRIL inhibitors may offer new hope to patients with IgAN.

## 1. Introduction

IgA nephropathy (IgAN) is the most common primary glomerulonephritis globally [1]. Approximately 30–40% of patients with IgAN progress to end-stage kidney disease (ESKD) within 20 years of the estimated time of disease onset [2,3]. Furthermore, the development of IgAN can reduce life expectancy by approximately 10 years [2]. Disease-specific treatments for IgAN are yet to be established. One reason is that the pathogenesis of IgAN remains unclear. Interactions between genetic predisposition and environmental factors determine susceptibility to IgAN [4]. Another reason is that the clinical and pathological manifestations of IgAN exhibit considerable heterogeneity among individuals and ethnicities [5]. Consequently, treatment modalities may vary among geographical regions. A tonsillectomy is considered an option in some regions, particularly in Japan, based on recent clinical studies. Some studies have reported that a tonsillectomy, either alone or in combination with steroid therapy, decreased hematuria and proteinuria while attenuating the progression of renal dysfunction [6,7]. Current guidelines recommend non-specific conservative management, including salt reduction, smoking cessation, blood pressure control, and the maximum tolerated dose of renin–angiotensin–aldosterone system inhibitors [8], which potentially worsen kidney function. Despite these interventions, there remains a notable residual risk of disease progression. The safety and efficacy of systemic corticosteroids for IgAN have been previously investigated multiple times [9,10]. However, there is currently insufficient evidence to support the recommendation of corticosteroids, owing to the persistent uncertainty about their benefits and risks. Therefore, optimal therapeutic strategies targeting the pathogenic mechanisms underlying IgAN to halt disease progression have been developed in recent years.

**Figure 1:**
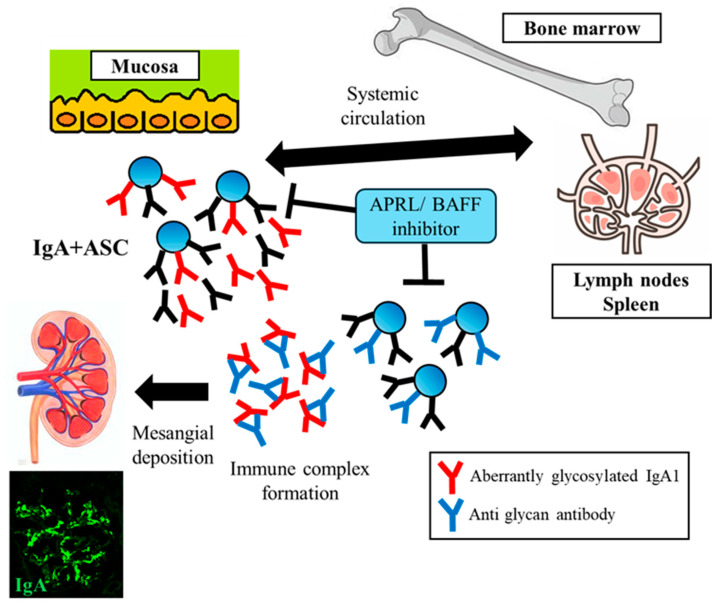
The proposed pathogenesis of IgA nephropathy and APRIL/BAFF-targeting therapy. After mucosal infection, class switching and recombination (CSR) of naïve B cells to IgA1 + B cells occurs via T-cell-dependent (TD) (cytokine-mediated) and T-cell-independent mechanisms (TID) (Toll-like receptor (TLR) ligation). A proliferation-inducing ligand (APRIL) and B-cell activating factor (BAFF) play key roles in driving both TD and TID CSR, resulting in the generation of IgA + B cells. The upregulation of these cytokines contributes to the production of aberrantly O–glycosylated IgA1 (Gd-IgA1) and the subsequent synthesis of autoantibodies directed against Gd-IgA1. During lymphocyte trafficking, some IgA antibody-secreting cells (ASCs) do not enter the systemic compartment. These displaced IgA ASCs migrate to systemic sites and produce Gd-IgA1, which is released into the systemic circulation. IgA1 secretion by the displaced mucosal ASCs is enhanced by TLR activation through mucosal-derived pathogen-associated molecular patterns that enter the systemic circulation. Some of the Gd-IgA1-containing immune complexes formed in circulation are deposited in the kidneys, where they activate mesangial cells, and induce glomerular injury.

Our understanding of the underlying complex autoimmune mechanisms has advanced in recent years. Aberrant O–glycosylated IgA1 (commonly termed galactose-deficient [Gd]-IgA1) and immune complexes formed by glycan-specific autoantibodies are crucial for the development of IgAN (Figure 1) [4,11]. Most human IgA is produced by plasma cells residing in mucosal-associated lymphoid tissues (MALTs). Two major regions are involved in IgAN, including gut-associated lymphoid tissues (GALTs) and nasopharynx-associated lymphoid tissue (NALT). Mucosae and lymphoid organs are regarded as sites of Gd-IgA1 production. Antigen-experienced IgA+ B cells leave MALTs and migrate to effector sites through the lymphatic system and circulation (Figure 1). It has been hypothesized that the dysregulated expression of homing receptors may redirect certain mucosa-derived B cells/plasma cells to the bone marrow instead of their intended mucosal destination in patients with IgAN [12,13] (Figure 1). Plasma cells with sustained production of IgA antibodies, derived from the mucosa or bone marrow, may produce and release Gd-IgA1 into circulation. Gd-IgA1-containing immune complexes are considered nephritogenic. Their deposition of immune complexes on mesangial cells stimulates the production of proinflammatory cytokines, resulting in the infiltration of inflammatory cells into the glomeruli, the proliferation of mesangial cells, and an uncontrolled inflammatory cascade via the activation of the complement pathway. Complement component 3 (C3) co-localizes with IgA in more than 90% of biopsies with IgAN, indicating complement activation. Complement activation may occur predominantly through the alternative and/or lectin pathways and serves as a central mediator of glomerular inflammation and damage [14]. These responses contribute to inflammation and scarring. Therefore, it has been hypothesized that Gd-IgA1 and Gd-IgA1-containing immune complexes are potential therapeutic targets. A reduction in the levels of these key molecules can potentially attenuate disease activity and improve kidney prognosis.

The tumor necrosis factor (TNF) family proliferation-inducing ligand (APRIL, also known as TNFSF13) plays a crucial role in regulating B cell activation, survival of long-lived plasma cells, and immunoglobulin isotype class switching [15,16,17]. This regulation is essential for maintaining effective immune responses and ensuring proper functioning of the immune system. B-cell activating factor of the TNF family (BAFF), which is closely associated with APRIL, acts as a pivotal survival factor for transitional and mature B cells. APRIL plays a pivotal role in the pathogenesis of IgAN, other autoimmune diseases such as lupus erythematosus and Sjögren’s syndrome [18], and some mature B cell neoplasms such as chronic lymphocytic leukemia and non-Hodgkin lymphoma [19,20]. BAFF is also thought to be involved in the pathogenesis of IgAN. As discussed in Section 3 on the role of APRIL in IgAN, emerging evidence indicates that APRIL is involved in the production of Gd-IgA1. Given the involvement of APRIL in the pathogenesis of IgAN, it is hypothesized that blocking the binding of APRIL to its receptors would potentially be a promising treatment option for IgAN that prevents downstream events such as the production of Gd-IgA1 and the subsequently formed Gd-IgA1 containing immune complexes, ultimately leading to the prevention of kidney damage and loss of function. Recent clinical trials have demonstrated the efficacy and safety of anti-APRIL and dual APRIL/BAFF treatments in patients with IgAN. Here, we review the physiological function of APRIL, the current knowledge on the role of APRIL in the pathogenesis of IgAN, and therapeutic strategies focusing on APRIL-targeting agents for IgAN.

## 2. Biology and Physiological Functions of APRIL

APRIL, which belongs to the TNF superfamily of ligands, is a type two transmembrane protein involved in late-stage B cell differentiation, generation and maintenance of antibody-secreting plasma cells, and IgA class switching [18]. Myeloid cells, including macrophages, dendritic cells, and blood monocytes, as well as polymorphonuclear cells, such as neutrophils, eosinophils, and mucosal epithelial cells, are responsible for APRIL production [21,22,23,24].

**Figure 2:**
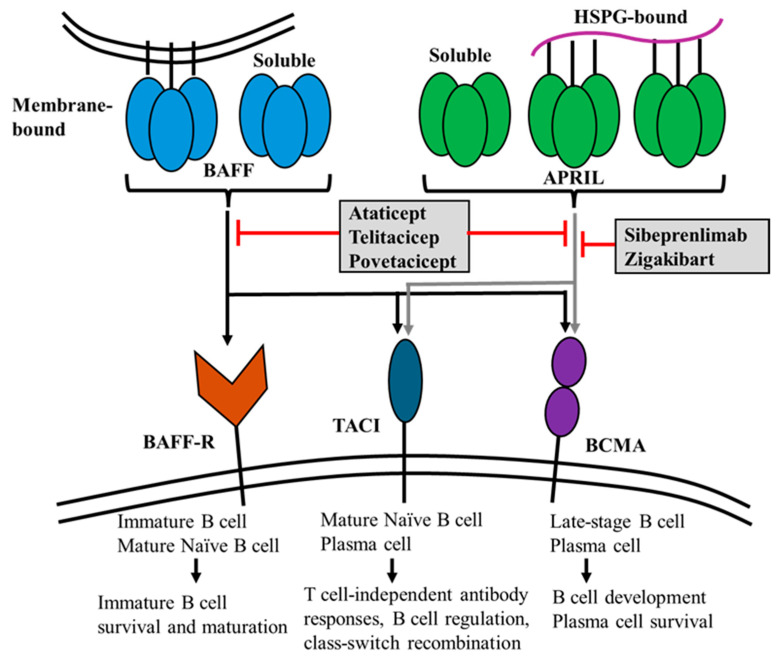
APRIL and BAFF signaling, their main physiological functions, and specifically targeted immunomodulatory agents. A proliferation-inducing ligand (APRIL) and B-cell activating factor (BAFF) both interact with two receptors: B-cell maturation antigen (BCMA) and transmembrane activator and calcium-modulator and cyclophilin-ligand interactor (TACI). BCMA is predominantly expressed on plasma cells, whereas TACI is expressed on both mature B cells and plasma cells. BAFF is distinct from APRIL due to its unique capacity to bind to the BAFF receptor (BAFF-R), which is predominantly found on both immature and mature naïve B cells. The heparan sulfate proteoglycan (HSPG), which serves as a receptor for APRIL, allows APRIL to interact with target cells, facilitating efficient signaling through TACI. Atacicept, telitacicept, and povetacicept are dual APRIL/BAFF antagonists, whereas sibeprenlimab and zigakibart exclusively inhibit APRIL signaling.

A related cytokine, B cell-activating factor from the TNF family (BAFF, also known as BLyS or TNFSF13B), has been identified as a crucial survival factor for B cells and is essential for their maturation. BAFF supports the survival of both transitional and mature B cells, plays a critical role in their maturation into immunoglobulin-producing cells, and contributes to their longevity. It has also been extensively studied, particularly in the field of autoimmunity. BAFF shares 48% homology with APRIL. APRIL and BAFF are produced as type II transmembrane proteins and are cleaved at the furin protease site, resulting in their secretion in a soluble form [25]. A recent study reported the expression of APRIL-δ isoforms lacking the consensus motif for the furin convertase, which remains membrane-bound on leukemia cell precursors [26]. BAFF exists in a membrane-bound state; however, its processed soluble form is essential for maintaining B cell homeostasis [27]. The homology between APRIL and BAFF also results in the sharing of these two receptors; however, BAFF possesses a unique receptor known as the BAFF receptor (BAFF-R; also known as TNFRSF13C) (Figure 2). APRIL and BAFF have two common receptors: the transmembrane activator and CAML interactor (TACI; also referred to as TNFRSF13B) and the B cell maturation antigen (BCMA; also known as TNFRSF17). APRIL exhibits a robust binding affinity to BCMA and moderate affinity to TACI, whereas BAFF exhibits a weak binding to BCMA and strong affinity to TACI [28]. Furthermore, BAFF has a high binding affinity for BAFF-R [28]. The three primary receptors, BAFF-R, TACI, and BCMA, exhibit unique expression patterns during the various stages of B cell development, reflecting their distinct functions. It has been hypothesized that APRIL and BAFF engage with their respective receptors through distinct mechanisms characterized by varying affinities [29]. Because of these subtle differences, targeting APRIL, BAFF, or both may be a therapeutic approach for other diseases caused by immune dysregulation. APRIL plays a pivotal role in mediating class switch recombination to IgA in T-cell-independent responses, primarily through its interaction with TACI (Table 1) [30]. In vitro experiments have demonstrated that recombinant APRIL can also enhance the T-cell-dependent switch [31]. There are two well-defined antibody-producing cells that represent the late differentiation stages of B lymphocytes: plasmablasts, generated within secondary lymphoid organs, and fully differentiated plasma cells, originating from plasmablasts. In a steady state, plasma cells migrate and become established in the bone marrow. Similar to autoimmune responses, this response can also be elicited in the presence of inflammation. In contrast to plasmablasts, plasma cells are characterized by their significant longevity. Myeloid-derived APRIL further supports plasma cell survival by promoting the formation of survival niches within the bone marrow and mucosal compartments, thereby establishing reservoirs for long-lived antibody-secreting cells. Plasma cell survival is primarily mediated by BCMA and TACI. While all plasma cells express BCMA, BCMA on these cells does not respond to BAFF stimulation [32]. BAFF-R exhibits an expression pattern closely resembling that of CD20, including the complete loss of expression during the plasma cell differentiation stage. BAFF-R is not expressed on B cell precursors within the bone marrow. However, it is expressed on immature B cells following the acquisition of a functional B cell receptor (BCR) [33]. BAFF-R is indispensable for the survival and maturation of immature B cells [34]. BAFF-R is also present in human mesangial cells and tubular epithelial cells (TECs) [35,36]. BAFF induces increased expression of BAFF-R in human tubular epithelial cells (TECs), possibly resulting in an autocrine loop [37]. TACI interacts with APRIL and heparan sulfate proteoglycans (HSPG) to exert its physiological effects [38]. BCMA expression is limited to plasmablasts and plasma cells [39], where it supports the survival of long-lived plasma cells.

## 3. The Role of APRIL in IgAN

Basic research, including biochemical, genetic, and physiological investigations, has suggested a potential role for APRIL in pathogenesis of IgAN. Genome-wide association studies (GWASs) identified a risk allele at the 17p23 TNFSF13 locus, which encodes APRIL [40]. Furthermore, a recent GWAS revealed that TACI, located on chromosome 17p11, is a novel susceptibility locus in addition to TNFSF13 [41]. A high serum level of APRIL was noted, which correlated with the urine protein/creatinine ratio (UPCR) and was observed to affect the prognosis of kidney function in patients with IgAN [42,43]. However, the organ and cellular origins of APRIL, the mechanisms underlying its overexpression, and its role in the pathogenesis of IgAN remain unknown. We have previously demonstrated that APRIL is overexpressed in tonsils, especially in germinal center (GC) B cells [44]. We also found that these GC B cells express not only the common cleavable APRILα mRNA but also the membrane-bound APRIL-δ/ζ mRNAs, which are less frequently expressed and not cleavable due to the lack of furin cleavage sites. APRIL-δ has also been identified as a novel APRIL isoform in patients with B cell precursor acute lymphoblastic leukemia [26,43]. Studies have demonstrated that mature B cell neoplasms, including chronic lymphocytic leukemia and follicular lymphoma, and B cells from patients with autoimmune diseases, such as systemic lupus erythematosus, generate APRIL [45,46,47]. This uncleavable membrane-bound variant of APRIL may persist on the B cell membrane for an extended period and enhance the synthesis of Gd IgA1, which is further accelerated by an excess of the soluble form of APRIL. Moreover, aberrant APRIL expression in tonsillar GC was correlated with clinical severity, and the patients exhibiting an APRIL overexpression in their tonsillar GC responded favorably to a tonsillectomy, which was accompanied by concurrent reductions in their serum levels of Gd-IgA1. We have also demonstrated that the TLR9 ligands CpG-oligonucleotides (CpG-ODN) increase gene expression and serum levels of APRIL in grouped ddY mice, which spontaneously develop IgAN, and that serum levels of APRIL are associated with those of Gd-IgA1 in these mice [48]. Moreover, in vitro experiments using human IgA1-secreting cell lines have shown that the knockdown of APRIL siRNA completely inhibits the overproduction of Gd-IgA1 [48]. The treatment of grouped ddY mice with an anti-APRIL antibody led to a reduction in IgA, aberrantly galactosylated IgA, and immune complexes in the serum, in addition to a reduction in proteinuria and markedly reduced kidney deposits of IgA, IgG, and C3, highlighting the potential therapeutic benefits of anti-APRIL agents for the treatment of IgAN [49]. However, to the best of our knowledge, no study has elucidated the role of APRIL in the direct modulation of IgA glycosylation. RNA sequencing and quantitative polymerase chain reaction (PCR) both showed that recombinant human TNFSF13 did not modulate the expression of glycosyltransferase enzymes in vitro using human B cells [50]. However, excess APRIL expression may contribute to a relative increase in the secretion of Gd-IgA1 by prolonging B cell survival [50].

Recently, the role of BAFF in IgAN has been discussed. Some studies reported elevated serum BAFF levels in patients with IgAN [42,43,51]. The serum levels of BAFF correlate with the severity of clinical data, such as the estimated glomerular filtration rate, serum creatinine levels, and histopathological findings of kidney biopsy, including the severity of tubular atrophy/interstitial fibrosis [51]. Furthermore, the stimulation of tonsillar mononuclear cells with CpG-ODNs resulted in the markedly higher production of IgA and BAFF in the patients with IgAN than in those without IgAN [52]. Transgenic mice overexpressing BAFF exhibited increased levels of polymeric IgA and mesangial IgA deposits, although extreme elevations in serum IgA levels were observed in these mice [42]. This condition depends on commensal flora and involves the disruption of the normal barrier between the mucosal and peripheral compartments [42]. An imbalance in the interactions between the immune system and commensal organisms contributes to the development of autoimmune disorders such as inflammatory bowel disease [53]. However, the treatment of grouped ddY mice with anti-BAFF agents failed to improve IgA and C3 deposition, urinary albumin excretion, serum levels of IgA-IgG immune complexes, and serum levels of Gd-IgA1 [54].

Preclinical studies in nonhuman primates have shown that treatment with sibeprenlimab (VIS649), a humanized IgG2 monoclonal antibody directed against APRIL, results in a dose-dependent decrease in serum IgA levels by up to 70%, highlighting the critical role of APRIL in regulating IgA production [49]. Treatment with belimumab, a monoclonal antibody targeting soluble BAFF, resulted in a modest 16% reduction in serum IgA levels, implying a restricted impact of BAFF on IgA production [55].

## 4. Current and Perspective Therapeutic Strategies Focusing on APRIL-Targeted Therapy

### 4.1. Systemic Corticosteroid Therapy and Targeting Gut-Associated Lymphoid Tissues (GALTs)

Clinical trials on the use of systemic corticosteroids for IgAN have been conducted over the years; however, their efficacy and safety remain controversial. However, the exact mechanism of action of corticosteroids in IgAN is not fully understood. Systemic corticosteroids have been thought to suppress the production of Gd-IgA1 and Gd-IgA1-containing immune complexes. However, the absence of a reduction in Gd-IgA1-positive plasmablasts/plasma cells in patients with IgAN treated with prednisone indicates that systemic corticosteroids may not effectively suppress Gd-IgA1 production. The efficacy and safety of systemic corticosteroids for the treatment of IgAN have shown variable results in multiple randomized controlled trials (RCTs). Two RCTs, Supportive Versus Immunosuppressive Therapy of Progressive IgA Nephropathy (STOP-IgAN) and Therapeutic Evaluation of Steroids in IgA Nephropathy Global (TESTING), which included a run-in period to optimize supportive care, did not demonstrate efficacy with complete safety [56,57]. The STOP-IgAN study did not show a significant improvement in the rate of estimated glomerular filtration rate (eGFR) decline with systemic corticosteroid administration in patients with IgAN, proteinuria above 0.75 g/day, and an eGFR greater than 60 mL/min/1.73 m^2^ [56]. In the TESTING trial, oral methylprednisolone was compared with placebo in patients with IgAN at a high risk of progression to renal failure, with a urinary protein >1 g/day and an eGFR of 20 to 120 mL/min. This study was terminated early because of a high incidence of adverse events such as serious infections in the corticosteroid group, although the corticosteroid group demonstrated a reduction in the composite endpoint, which include a reduction of over 40% in eGFR and the incidence of end-stage renal failure [57]. Although the TESTING II study found that tapering methylprednisolone (0.4 mg/kg/day over six months) slowed the decline of kidney function in patients with IgAN, this treatment also led to more adverse events [9]. In contrast, a survey involving a Japanese cohort confirmed that the incidence of adverse events such as severe infections and hyperglycemia due to corticosteroid use was low [58]. The corticosteroid doses commonly used in Japan are lower than those used in clinical trials.

The local immunosuppressive effect of the targeted-release formulation (TRF) budesonide (Nefecon; Calliditas Therapeutics AB) on the intestinal mucosa may be a promising novel therapeutic candidate for IgAN. Macroscopic hematuria due to upper respiratory tract infections, such as tonsillitis or intestinal tract infections, highlights the involvement of mucosal–kidney connections in the pathogenesis of IgAN [5]. The interaction between the dysregulated immune system in the GALT and IgAN has been a topic of discussion for decades [59,60]. Recent GWASs have identified common risk alleles for IgAN and inflammatory bowel disease (IBD) that are linked to the integrity of the intestinal mucosal barrier [61,62]. Population-based cohort studies have shown that patients with IgAN have an increased risk of developing IBD [63,64]. The gut–kidney axis hypothesis highlights the pivotal role of the imbalance between the immune system and microbiota in the development and progression of IgAN. In patients with IgAN, there is an increase in the serum levels of IgA1, which lacks galactose in its hinge region. This deficiency exposes the N-acetylgalactosamine (GalNAc) residues that are recognized by anti-glycan autoantibodies. The gut microbiota can induce B cell activation via BAFF, and this enhanced stimulation promotes a significant transition from IgA2 to IgA1 production [65]. Notably, high serum levels of gut-homing IgA + B lymphocytes indicates the involvement of intestinal mucosal immune dysregulation in the pathogenesis of IgAN [43]. Budesonide, a novel oral glucocorticoid TRF, was specifically designed to be deployed in the ileum and to deliver glucocorticoids locally to the ileal gut-associated lymphoid system with limited systemic exposure. The global phase 3 NefIgArd trial demonstrated that treatment with Nefecon at 16 mg/day significantly reduced the decline in eGFR and proteinuria in patients with IgAN who were already on an optimized RAS blockade, implying a disease-modifying effect of Nefecon in these patients [66]. Budesonide is recognized for its favorable safety profile compared to systemic corticosteroids, primarily because of its significant hepatic first-pass metabolism, resulting in less than 10% of the drug entering the systemic circulation [67]. Importantly, this study demonstrated a reduction in the serum levels of Gd-IgA1 and IgA/IgG immune complexes. Reduction in the serum levels of APRIL, BAFF, and soluble BCMA were observed following treatment with Nefecon, indicating an impact on disease activity through the direct modulation of APRIL and BAFF [68]. This study highlights the changes in chemokines related to lymphocyte migration to the intestinal mucosa, suggesting that Nefecon impacts immune cells residing in or migrating to gut-associated lymphoid tissues (GALTs). Nefecon received an accelerated approval from the United States Food and Drug Administration (FDA) in December 2021 for the treatment of IgAN. In December 2023, the FDA granted a full approval to Nefecon, making it the first fully approved treatment for IgAN.

### 4.2. B-Cell and Plasma Cell-Targeted Treatment

Targeting B cells, which are responsible for the production of Gd-IgA1, has recently emerged as a promising strategy for IgAN treatment. Rituximab, a monoclonal anti-CD20 antibody, does not significantly affect urinary protein levels, eGFR, or Gd-IgA1 levels, despite the effective depletion of peripheral B cells [69]. Plasmablasts are rapidly produced as short-lived effector cells that home to either the bone marrow or the mucosa, where they secrete antibodies as terminally differentiated plasma cells. CD20 is highly expressed in mature naïve B cells but is gradually lost during B cell differentiation. As B cells differentiate into plasma cells, plasmablasts and plasma cells lose their CD20 expression [70]. Notably, IgA plasmablasts have been identified in the mucosa following rituximab treatment [71]. Therefore, anti-CD20 treatment may not reduce the number of plasmablasts or plasma cells responsible for the production of Gd-IgA1, thereby perpetuating Gd-IgA1 synthesis even after rituximab treatment in patients with IgAN. An alternative depletion approach currently under evaluation for IgAN and adapted for the treatment of multiple myeloma is CD38 depletion (Table 2). In contrast to B cells, plasma cells exhibit high CD38 expression and low CD20 expression [72]. BAFF has been demonstrated to selectively augment the survival and differentiation of CD38 + plasmablasts derived from activated human memory B cells. Strategies targeting CD38 + plasma cells, such as felzartamab, a human IgG1 monoclonal antibody, are being explored in phase 2 RCTs for their potential to target the cells responsible for the production of Gd-IgA1 (NCT05065970). Mezagitamab, another anti-CD38 human IgG1 monoclonal antibody, is currently being evaluated in phase 1 studies in IgAN (NCT05174221). The proteasome inhibitor bortezomib, a plasma cell-depleting agent, has been used to treat multiple myeloma by inhibiting the transcriptional factor nuclear facto-kappa B (NF-κB) and inducing the apoptosis of myeloma cells through a misfolded protein response. An open pilot study involving eight patients with IgAN was conducted to evaluate the effects of bortezomib [73]. The results showed that three of the eight patients achieved complete remission after four doses of bortezomib at 1 year of follow-up, suggesting that plasma cell depletion could potentially improve outcomes in IgAN. Therefore, larger trials are needed to confirm the efficacy and safety of these drugs. 

Based on multiple studies, strategies for APRIL inhibition are currently being explored as potential therapies for IgAN (Table 2). Several novel agents targeting APRIL, including sibeprenlimab, atacicept, telitacicept, povetacicept, and zigakibart are currently being investigated for the treatment of IgAN (Figure 2). These novel treatments exert their therapeutic effects by reducing the production of Gd-IgA1 and the subsequently formed Gd-IgA1-containing immune complexes (Figure 1). Preliminary results suggest that these agents are well-tolerated and reduce Gd-IgA1 levels, leading to an improvement in proteinuria. A phase 2 multicenter double-blind RCT assessed the efficacy of 12 monthly intravenous infusions of sibeprenlimab at doses of 2, 4, or 8 mg/kg body weight versus placebo in patients with IgAN who exhibited a high susceptibility to disease progression [74]. At the end of the 12-month period, the sibeprenlimab cohort showed significantly greater reductions in the 24-h UPCR from the baseline, in a dose-dependent manner, compared to the placebo group. A significant reduction in the rate of eGFR decline was observed in the two higher-dose groups. Importantly, these effects were accompanied by a decrease in the serum levels of APRIL and Gd-IgA1 in the treatment group, although there was no evidence of clinically significant immunosuppression. The decreased UPCR observed in the sibeprenlimab group continued for five months after the treatment was discontinued. However, the APRIL and Gd-IgA1 levels tended to increase, suggesting the need for ongoing therapeutic interventions. Phase 3 trials and open-label extension studies are currently underway (NCT05248646 and NCT05248659).

Blisibimod, a monoclonal antibody against both soluble and membrane-bound BAFF, was one of the first agents approved for the treatment of systemic lupus erythematosus (SLE). Clinical studies have demonstrated benefits, such as steroid reduction, decreased proteinuria, and improvements in serological markers in patients with SLE [75]. Blisibimod has been evaluated for its efficacy and safety in patients with IgAN. The phase 2/3 BRIGHT-SC study (NCT02062684) demonstrated that blisibimod-mediated BAFF inhibition significantly reduced peripheral B cells, immunoglobulins, and UPCR in patients with IgAN compared to a placebo [76]. The long-term efficacy and safety of targeting BAFF alone in IgAN remains uncertain because comprehensive results from relevant studies have not yet been published.

Atacicept is a novel immunomodulatory agent composed of a fully humanized recombinant fusion protein that includes the Fc region of human IgG1 and the TACI-binding domain [77,78]. Atacicept acts by binding soluble BAFF and APRIL and membrane-bound BAFF, thereby interfering with the cellular interactions of these cytokines and their receptors, TACI, BCMA, and BAFF-R. In a phase 2 study, atacicept dose-dependently reduced proteinuria and Gd-IgA1 antibody levels [78]. The phase 2B ORIGIN clinical trial of atacicept, involving 116 patients with IgAN, also showed promising results. The trial met its primary endpoint with the 150-mg-dose group achieving a 33% mean reduction in proteinuria from the baseline at 24 weeks [79]. Notably, atacicept treatment resulted in significant dose-dependent reductions in IgA, IgG, IgM, and Gd-IgA1 levels by week 24, which were sustained until week 72. The safety profile of atacicept was similar to that of the placebo. Additionally, the 72-week data indicated a consistent and sustained reduction in proteinuria and a stabilization of eGFR, further supporting the potential of atacicept as a disease-modifying treatment for IgAN. A phase 3 ORIGIN study is currently evaluating the efficacy and safety of a 150 mg dose of atacicept compared to a placebo in terms of urinary findings, kidney function, and immunomodulatory effects in patients with IgAN (NCT04716231).

Telitacicept, a recombinant fusion protein consisting of TACI and the Fc portion of human IgG, inhibits both APRIL and BAFF. Based on prior efficacy and safety data, telitacicept received approval in China for the management of patients with active SLE [80,81]. A phase 2 RCT showed that 240 mg of telitacicept reduced the mean 24-h proteinuria by 49%, and stabilized the mean eGFR at 24 weeks in 44 Chinese patients with IgAN [82]. In addition, treatment with 160 or 240 mg of telitacicept decreased circulatory Gd-IgA1 and IgA-containing immune complexes at week 24 [83]. These findings suggest that telitacicept reduces the risk of disease progression. A phase 3 trial is currently in progress (NCT05799287).

Povetacicept, a human TACI–Fc fusion protein engineered for dual APRIL/BAFF inhibition, has been studied in patients with IgAN, primary membranous nephropathy (MN), and lupus nephritis. A phase 1B/2A study (NCT05732402) recruited patients with autoimmune glomerulonephritis, including IgAN. Preliminary data presented in 2024 at the World Congress of Nephrology (WCN) indicate that treatment with povetacicept every 4 weeks is associated with a significant reduction in proteinuria, stable kidney function, and significant reductions in circulating Gd-IgA1 levels in patients with IgAN [84].

Zigakibart (BION-1301) is a humanized IgG4 monoclonal antibody that blocks APRIL. The interim results from a phase 1/2 trial of zigakibart in patients with IgAN (NCT03945318) demonstrated clinically meaningful and sustained reductions in proteinuria, accompanied by rapid and sustained decreases in serum levels of IgA and Gd-IgA1 [85,86,87]. Zigakibart was well-tolerated, with no severe adverse effects reported. The BEYOND study, a phase 3, randomized, double-blind, placebo-controlled trial to explore the safety and efficacy of zigakibart in patients with IgAN who are at risk of progressing renal dysfunction is currently in progress (NCT05852938).

Although APRIL/BAFF inhibition has shown promising results with no notable adverse events, the effects of suppressing one on the expression of the other are not fully understood. Some studies have suggested that targeting one of these cytokines may not necessarily lead to the overexpression of the other [88,89]. However, further research is required to clarify this interaction and its implications in immune regulation and potential therapies.

## 5. Conclusions and Future Perspectives

Our recent understanding of the pathogenesis of IgAN including immune dysregulation has evolved, and various strategies that focus on inhibiting signaling pathways including APRIL/BAFF, depleting plasma cells, modulating mucosal immunity, blocking complement cascades, and other downstream pathways that are activated following IgA deposition to treat different aspects of the pathogenesis of IgAN and the progression, have shown safety and efficacy. However, the appropriate indication or optimal treatment duration with each modulator, including APRIL/BAFF inhibitors, remains unclear. The optimal choice between exclusive APRIL antagonists and combined APRIL/BAFF inhibitors, in terms of safety and efficacy, remains uncertain. Moreover, we may need to consider the nephritogenic mucosal site of each patient based on their clinical characteristics or ethnicity, depending on the treatment options. Further validation studies are required to resolve these issues.

## Figures and Tables

**Table 1:** Representative physiological functions of a proliferation-inducing ligand (APRIL) and B-cell activating factor of the TNF family (BAFF).

Cytokine	Mediated by	Function
APRIL	TACI	B cell isotype switching
		T-cell-dependent and -independent antibody response
		Differentiation of B cell to plasmablast
		Plasma cell and long-lived plasma cell survival
		Production of antiapoptotic protein
	BCMA	Plasmablast and plasma cell survival
BAFF	BAFF-R	B cell survival and maturation
	TACI	Regulation of B cell survival and proliferation
		T-cell-independent antibody response
		Promotion of long-lived plasma cell survival
		Class switch recombination induction
	BCMA	B cell development

TACI, transmembrane activator and calcium-modulator and cyclophilin-ligand interactor; BCMA, B-cell maturation antigen; BAFF-R, the BAFF receptor.

**Table 2:** Recently completed, ongoing, or upcoming clinical trials of anti-plasma cell, and anti-APRIL/BAFF therapies in IgA nephropathy.

Agent	Clinical Trial Name	Phase	Target	Registration No.	Route of Administration
Felzartamab	IGNAZ	II	CD38	NCT05065970	IV (depending on the arms)
Mezagitamab		I	CD38	NCT05174221	Weekly SC
Bortezomib		II	Proteasome	NCT05383547	IV (monthly cycle)
Sibeprenlimab	ENVISION	II	APRIL	NCT04287985	4-weekly IV
	VISIONARY	III	APRIL	NCT05248646	4-weekly SC
		II/III	APRIL	NCT05248659	4-weekly SC (single-arm, open-label)
Ataticept	JANUS	II	APRIL + BAFF	NCT02808429	Weekly SC
	ORIGIN	IIb	APRIL + BAFF	NCT04716231	Weekly SC
Telitacicept		II	APRIL + BAFF	NCT04905212	4-weekly SC
		III	APRIL + BAFF	NCT05799287	Weekly SC /2-weekly SC
Zigakibart	ADU-CL-19	I/II	APRIL	NCT03945318	2-weekly IV /2-weekly SC
	BEYOND	III	APRIL	NCT05852938	2-weekly SC
Povetacicept	RUBY-3	Ib/IIa	APRIL + BAFF	NCT05732402	4-weekly SC

APRIL, a proliferation-inducing ligand (APRIL); BAFF, B cell-activating factor (BAFF); IV, intravenous; SC, subcutaneous.

## Data Availability

Not applicable.

## References

[1] McGrogan A., Franssen C.F.M., de Vries C.S. (2010). The incidence of primary glomerulonephritis worldwide: A systematic review of the literature. Nephrol. Dial. Transplant..

[2] Hastings M.C., Bursac Z., Julian B.A., Baca E.V., Featherston J., Woodford S.Y., Bailey L., Wyatt R.J. (2017). Life Expectancy for Patients From the Southeastern United States With IgA Nephropathy. Kidney Int. Rep..

[3] Moriyama T., Tanaka K., Iwasaki C., Oshima Y., Ochi A., Kataoka H., Itabashi M., Takei T., Uchida K., Nitta K. (2014). Prognosis in IgA Nephropathy: 30-Year Analysis of 1,012 Patients at a Single Center in Japan. PLoS ONE.

[4] Suzuki H., Kiryluk K., Novak J., Moldoveanu Z., Herr A.B., Renfrow M.B., Wyatt R.J., Scolari F., Mestecky J., Gharavi A.G. (2011). The Pathophysiology of IgA Nephropathy. J. Am. Soc. Nephrol..

[5] Suzuki Y., Monteiro R.C., Coppo R., Suzuki H. (2021). The Phenotypic Difference of IgA Nephropathy and its Race/Gender-dependent Molecular Mechanisms. Kidney360.

[6] Hirano K., Matsuzaki K., Yasuda T., Nishikawa M., Yasuda Y., Koike K., Maruyama S., Yokoo T., Matsuo S., Kawamura T. (2019). Association Between Tonsillectomy and Outcomes in Patients With Immunoglobulin A Nephropathy. JAMA Netw. Open.

[7] Koike K., Kawamura T., Hirano K., Nishikawa M., Shimizu A., Joh K., Katafuchi R., Hashiguchi A., Yano Y., Matsuzaki K. (2023). Clinicopathological prognostic stratification for proteinuria and kidney survival in IgA nephropathy: A Japanese prospective cohort study. Clin. Kidney J..

[8] Rovin B.H., Adler S.G., Barratt J., Bridoux F., Burdge K.A., Chan T.M., Cook H.T., Fervenza F.C., Gibson K.L., Glassock R.J. (2021). KDIGO 2021 Clinical practice guideline for the management of glomerular diseases. Kidney Int..

[9] Lv J., Wong M.G., Hladunewich M.A., Jha V., Hooi L.S., Monaghan H., Zhao M., Barbour S., Jardine M.J., Reich H.N. (2022). Effect of oral methylprednisolone on decline in kidney function or kidney failure in patients with IgA nephropathy: The TESTING randomized clinical trial. JAMA.

[10] Zhang Y.-M., Lv J.-C., Wong M.G., Zhang H., Perkovic V. (2023). Glucocorticoids for IgA nephropathy-pro. Kidney Int..

[11] Cheung C.K., Barratt J. (2022). Further Evidence for the Mucosal Origin of Pathogenic IgA in IgA Nephropathy. J. Am. Soc. Nephrol..

[12] Knoppova B., Reily C., Maillard N., Rizk D.V., Moldoveanu Z., Mestecky J., Raska M., Renfrow M.B., Julian B.A., Novak J. (2016). The Origin and Activities of IgA1-Containing Immune Complexes in IgA Nephropathy. Front. Immunol..

[13] Gesualdo L., Di Leo V., Coppo R. (2021). The mucosal immune system and IgA nephropathy. Semin. Immunopathol..

[14] Rajasekaran A., Julian B.A., Rizk D.V. (2020). IgA Nephropathy: An Interesting Autoimmune Kidney Disease. Am. J. Med. Sci..

[15] Sakurai D., Hase H., Kanno Y., Kojima H., Okumura K., Kobata T. (2006). TACI regulates IgA production by APRIL in collaboration with HSPG. Blood.

[16] He B., Santamaria R., Xu W., Cols M., Chen K., Puga I., Shan M., Xiong H., Bussel J.B., Chiu A. (2010). The transmembrane activator TACI triggers immunoglobulin class switching by activating B cells through the adaptor MyD88. Nat. Immunol..

[17] Belnoue E., Pihlgren M., McGaha T.L., Tougne C., Rochat A.-F., Bossen C., Schneider P., Huard B., Lambert P.-H., Siegrist C.-A. (2008). APRIL is critical for plasmablast survival in the bone marrow and poorly expressed by early-life bone marrow stromal cells. Blood.

[18] Baert L., Manfroi B., Casez O., Sturm N., Huard B. (2018). The role of APRIL-A proliferation inducing ligand-In autoimmune diseases and expectations from its targeting. J. Autoimmun..

[19] Kern C., Cornuel J.-F., Billard C., Tang R., Rouillard D., Stenou V., Defrance T., Ajchenbaum-Cymbalista F., Simonin P.-Y., Feldblum S. (2004). Involvement of BAFF and APRIL in the resistance to apoptosis of B-CLL through an autocrine pathway. Blood.

[20] Chiu A., Xu W., He B., Dillon S.R., Gross J.A., Sievers E., Qiao X., Santini P., Hyjek E., Lee J.-W. (2006). Hodgkin lymphoma cells express TACI and BCMA receptors and generate survival and proliferation signals in response to BAFF and APRIL. Blood.

[21] Hardenberg G., Planelles L., Schwarte C.M., van Bostelen L., Le Huong T., Hahne M., Medema J.P. (2007). Specific TLR ligands regulate APRIL secretion by dendritic cells in a PKR-dependent manner. Eur. J. Immunol..

[22] Chu V.T., Fröhlich A., Steinhauser G., Scheel T., Roch T., Fillatreau S., Lee J.J., Löhning M., Berek C. (2011). Eosinophils are required for the maintenance of plasma cells in the bone marrow. Nat. Immunol..

[23] Matthes T., Dunand-Sauthier I., Santiago-Raber M.-L., Krause K.-H., Donze O., Passweg J., McKee T., Huard B. (2011). Production of the plasma-cell survival factor a proliferation-inducing ligand (APRIL) peaks in myeloid precursor cells from human bone marrow. Blood.

[24] Puga I., Cols M., Barra C.M., He B., Cassis L., Gentile M., Comerma L., Chorny A., Shan M., Xu W. (2011). B cell–helper neutrophils stimulate the diversification and production of immunoglobulin in the marginal zone of the spleen. Nat. Immunol..

[25] López-Fraga M., Fernández R., Albar J.P., Hahne M. (2001). Biologically active APRIL is secreted following intracellular processing in the Golgi apparatus by furin convertase. Embo Rep..

[26] Maia S., Pelletier M., Ding J., Hsu Y.-M., Sallan S.E., Rao S.P., Nadler L.M., Cardoso A.A. (2011). Aberrant Expression of Functional BAFF-System Receptors by Malignant B-Cell Precursors Impacts Leukemia Cell Survival. PLoS ONE.

[27] Bossen C., Tardivel A., Willen L., Fletcher C.A., Perroud M., Beermann F., Rolink A.G., Scott M.L., Mackay F., Schneider P. (2010). Mutation of the BAFF furin cleavage site impairs B-cell homeostasis and antibody responses. Eur. J. Immunol..

[28] Day E.S., Cachero T.G., Qian F., Sun Y., Wen D., Pelletier M., Hsu Y.-M., Whitty A. (2005). Selectivity of BAFF/BLyS and APRIL for Binding to the TNF Family Receptors BAFFR/BR3 and BCMA. Biochemistry.

[29] Mackay F., Schneider P., Rennert P., Browning J. (2003). BAFF and APRIL: A Tutorial on B Cell Survival. Annu. Rev. Immunol..

[30] Castigli E., Wilson S.A., Scott S., Dedeoglu F., Xu S., Lam K.-P., Bram R.J., Jabara H., Geha R.S. (2005). TACI and BAFF-R mediate isotype switching in B cells. J. Exp. Med..

[31] Castigli E., Wilson S.A., Elkhal A., Ozcan E., Garibyan L., Geha R.S. (2007). Transmembrane activator and calcium modulator and cyclophilin ligand interactor enhances CD40-driven plasma cell differentiation. J. Allergy Clin. Immunol..

[32] Bossen C., Cachero T.G., Tardivel A., Ingold K., Willen L., Dobles M., Scott M.L., Maquelin A., Belnoue E., Siegrist C.-A. (2008). TACI, unlike BAFF-R, is solely activated by oligomeric BAFF and APRIL to support survival of activated B cells and plasmablasts. Blood.

[33] Mihalcik S.A., Huddleston P.M., Wu X., Jelinek D.F. (2010). The structure of the TNFRSF13C promoter enables differential expression of BAFF-R during B cell ontogeny and terminal differentiation. J. Immunol..

[34] Ng L.G., Sutherland A.P.R., Newton R., Qian F., Cachero T.G., Scott M.L., Thompson J.S., Wheway J., Chtanova T., Groom J. (2004). B Cell-Activating Factor Belonging to the TNF Family (BAFF)-R Is the Principal BAFF Receptor Facilitating BAFF Costimulation of Circulating T and B Cells. J. Immunol..

[35] Cao Y., Lu G., Chen X., Chen X., Guo N., Li W. (2020). BAFF is involved in the pathogenesis of IgA nephropathy by activating the TRAF6/NF-κB signaling pathway in glomerular mesangial cells. Mol. Med. Rep..

[36] Zheng N., Wang D., Ming H., Zhang H., Yu X. (2015). BAFF promotes proliferation of human mesangial cells through interaction with BAFF-R. BMC Nephrol..

[37] Schwarting A., Relle M., Meineck M., Föhr B., Triantafyllias K., Weinmann A., Roth W., Weinmann-Menke J. (2017). Renal tubular epithelial cell-derived BAFF expression mediates kidney damage and correlates with activity of proliferative lupus nephritis in mouse and men. Lupus.

[38] Moreaux J., Sprynski A., Dillon S.R., Mahtouk K., Jourdan M., Ythier A., Moine P., Robert N., Jourdan E., Rossi J.F. (2009). APRIL and TACI interact with syndecan-1 on the surface of multiple myeloma cells to form an essential survival loop. Eur. J. Haematol..

[39] Stadanlick J.E., Kaileh M., Karnell F.G., Scholz J.L., Miller J.P., Quinn W.J., Brezski R.J., Treml L.S., Jordan K.A., Monroe J.G. (2008). Tonic B cell antigen receptor signals supply an NF-kappaB substrate for prosurvival BLyS signaling. Nat. Immunol..

[40] Yu X.-Q., Li M., Zhang H., Low H.-Q., Wei X., Wang J.-Q., Sun L.-D., Sim K.-S., Li Y., Foo J.-N. (2011). A genome-wide association study in Han Chinese identifies multiple susceptibility loci for IgA nephropathy. Nat. Genet..

[41] Kiryluk K., Sanchez-Rodriguez E., Zhou X.-J., Zanoni F., Liu L., Mladkova N., Khan A., Marasa M., Zhang J.Y., Balderes O. (2023). Genome-wide association analyses define pathogenic signaling pathways and prioritize drug targets for IgA nephropathy. Nat. Genet..

[42] McCarthy D.D., Kujawa J., Wilson C., Papandile A., Poreci U., Porfilio E.A., Ward L., Lawson M.A., Macpherson A.J., McCoy K.D. (2011). Mice overexpressing BAFF develop a commensal flora-dependent, IgA-associated nephropathy. J. Clin. Investig..

[43] Sallustio F., Curci C., Chaoul N., Fontò G., Lauriero G., Picerno A., Divella C., Di Leo V., De Angelis M., Ben Mkaddem S. (2021). High levels of gut-homing immunoglobulin A + B lymphocytes support the pathogenic role of intestinal mucosal hyperresponsiveness in immunoglobulin A nephropathy patients. Nephrol. Dial. Transplant..

[44] Muto M., Manfroi B., Suzuki H., Joh K., Nagai M., Wakai S., Righini C., Maiguma M., Izui S., Tomino Y. (2016). Toll-Like Receptor 9 Stimulation Induces Aberrant Expression of a Proliferation-Inducing Ligand by Tonsillar Germinal Center B Cells in IgA Nephropathy. J. Am. Soc. Nephrol..

[45] He B., Chadburn A., Jou E., Schattner E.J., Knowles D.M., Cerutti A. (2004). Lymphoma B Cells Evade Apoptosis through the TNF Family Members BAFF/BLyS and APRIL. J. Immunol..

[46] Gupta M., Dillon S.R., Ziesmer S.C., Feldman A.L., Witzig T.E., Ansell S.M., Cerhan J.R., Novak A.J. (2009). A proliferation-inducing ligand mediates follicular lymphoma B-cell proliferation and cyclin D1 expression through phosphatidylinositol 3-kinase–regulated mammalian target of rapamycin activation. Blood.

[47] Chu V.T., Enghard P., Schürer S., Steinhauser G., Rudolph B., Riemekasten G., Berek C. (2009). Systemic activation of the immune system induces aberrant BAFF and APRIL expression in B cells in patients with systemic lupus erythematosus. Arthritis Rheum..

[48] Makita Y., Suzuki H., Kano T., Takahata A., Julian B.A., Novak J., Suzuki Y. (2019). TLR9 activation induces aberrant IgA glycosylation via APRIL- and IL-6–mediated pathways in IgA nephropathy. Kidney Int..

[49] Myette J.R., Kano T., Suzuki H., Sloan S.E., Szretter K.J., Ramakrishnan B., Adari H., Deotale K.D., Engler F., Shriver Z. (2019). A Proliferation Inducing Ligand (APRIL) targeted antibody is a safe and effective treatment of murine IgA nephropathy. Kidney Int..

[50] Han S.S., Yang S.H., Choi M., Kim H.-R., Kim K., Lee S., Moon K.C., Kim J.Y., Lee H., Lee J.P. (2016). The Role of TNF Superfamily Member 13 in the Progression of IgA Nephropathy. J. Am. Soc. Nephrol..

[51] Xin G., Shi W., Xu L.-X., Su Y., Yan L.-J., Li K.-S. (2012). Serum BAFF is elevated in patients with IgA nephropathy and associated with clinical and histopathological features. J. Nephrol..

[52] Goto T., Bandoh N., Yoshizaki T., Nozawa H., Takahara M., Ueda S., Hayashi T., Harabuchi Y. (2008). Increase in B-cell-activation factor (BAFF) and IFN-gamma productions by tonsillar mononuclear cells stimulated with deoxycytidyl-deoxyguanosine oligodeoxynucleotides (CpG-ODN) in patients with IgA nephropathy. Clin. Immunol..

[53] Xavier R.J., Podolsky D.K. (2007). Unravelling the pathogenesis of inflammatory bowel disease. Nature.

[54] Kim J., Suzuki H., Kano T., Fukao Y., Nakayama M., Suzuki Y. (2022). POS-399 Anti-BAFF antibody is effective to inhibit the production of immunoglobulins, but not nephritogenic IgA in murine IgA nephropathy. Kidney Int. Rep..

[55] Navarra S.V., Guzmán R.M., E Gallacher A., Hall S., A Levy R., E Jimenez R., Li E.K.-M., Thomas M., Kim H.-Y., León M.G. (2011). Efficacy and safety of belimumab in patients with active systemic lupus erythematosus: A randomised, placebo-controlled, phase 3 trial. Lancet.

[56] Rauen T., Eitner F., Fitzner C., Sommerer C., Zeier M., Otte B., Panzer U., Peters H., Benck U., Mertens P.R. (2015). Intensive Supportive Care plus Immunosuppression in IgA Nephropathy. N. Engl. J. Med..

[57] Lv J., Zhang H., Wong M.G., Jardine M.J., Hladunewich M., Jha V., Monaghan H., Zhao M., Barbour S., Reich H. (2017). Effect of oral methylprednisolone on clinical outcomes in patients with IgA nephropathy: The TESTING randomized clinical trial. JAMA.

[58] Matsuzaki K., Suzuki H., Kikuchi M., Koike K., Komatsu H., Takahashi K., Narita I., Okada H. (2023). Committee of Clinical Practical Guideline for IgA Nephropathy 2020 Current treatment status of IgA nephropathy in Japan: A questionnaire survey. Clin. Exp. Nephrol..

[59] Barratt J., Rovin B.H., Cattran D., Floege J., Lafayette R., Tesar V., Trimarchi H., Zhang H. (2020). Why target the gut to treat IgA nephropathy?. Kidney Int. Rep..

[60] Coppo R. (2018). The Gut-Renal Connection in IgA Nephropathy. Semin. Nephrol..

[61] Kiryluk K., Li Y., Scolari F., Sanna-Cherchi S., Choi M., Verbitsky M., Fasel D., Lata S., Prakash S., Shapiro S. (2014). Discovery of new risk loci for IgA nephropathy implicates genes involved in immunity against intestinal pathogens. Nat. Genet..

[62] Shi D., Zhong Z., Wang M., Cai L., Fu D., Peng Y., Guo L., Mao H., Yu X., Li M. (2019). Identification of susceptibility locus shared by IgA nephropathy and inflammatory bowel disease in a Chinese Han population. J. Hum. Genet..

[63] Rehnberg J., Symreng A., Ludvigsson J.F., Emilsson L. (2021). Inflammatory Bowel Disease Is More Common in Patients with IgA Nephropathy and Predicts Progression of ESKD: A Swedish Population-Based Cohort Study. J. Am. Soc. Nephrol..

[64] Nakayama T., Kaneko H., Okada A., Suzuki Y., Fujiu K., Takeda N., Morita H., Takeda N., Fukui A., Yokoo T. (2024). Association of inflammatory bowel disease with incident immunoglobulin A nephropathy. Clin. J. Am. Soc. Nephrol..

[65] Brandtzaeg P., Carlsen H.S., Halstensen T.S. (2006). The B-cell system in inflammatory bowel disease. Adv. Exp. Med. Biol..

[66] Lafayette R., Kristensen J., Stone A., Floege J., Tesař V., Trimarchi H., Zhang H., Eren N., Paliege A., Reich H.N. (2023). Efficacy and safety of a targeted-release formulation of budesonide in patients with primary IgA nephropathy (NefIgArd): 2-year results from a randomised phase 3 trial. Lancet.

[67] Edsbäcker S., Andersson T. (2004). Pharmacokinetics of Budesonide (Entocort^TM^ EC) Capsules for Crohn’s Disease. Clin. Pharmacokinet..

[68] Wimbury D., Muto M., Bhachu J.S., Scionti K., Brown J., Molyneux K., Seikrit C., Maixnerová D., Pérez-Alós L., Garred P. (2024). Targeted-release budesonide modifies key pathogenic biomarkers in immunoglobulin A nephropathy: Insights from the NEFIGAN trial. Kidney Int..

[69] Lafayette R.A., Canetta P.A., Rovin B.H., Appel G.B., Novak J., Nath K.A., Sethi S., Tumlin J.A., Mehta K., Hogan M. (2016). A Randomized, Controlled Trial of Rituximab in IgA Nephropathy with Proteinuria and Renal Dysfunction. J. Am. Soc. Nephrol..

[70] Jourdan M., Caraux A., Caron G., Robert N., Fiol G., Rème T., Bolloré K., Vendrell J.-P., Le Gallou S., Mourcin F. (2011). Characterization of a Transitional Preplasmablast Population in the Process of Human B Cell to Plasma Cell Differentiation. J. Immunol..

[71] Mei H.E., Frölich D., Giesecke C., Loddenkemper C., Reiter K., Schmidt S., Feist E., Daridon C., Tony H.P., Radbruch A. (2010). Steady-state generation of mucosal IgA+ plasmablasts is not abrogated by B-cell depletion therapy with rituximab. Blood.

[72] Schrezenmeier E., Jayne D., Dörner T. (2018). Targeting B Cells and Plasma Cells in Glomerular Diseases: Translational Perspectives. J. Am. Soc. Nephrol..

[73] Hartono C., Chung M., Perlman A.S., Chevalier J.M., Serur D., Seshan S.V., Muthukumar T. (2018). Bortezomib for Reduction of Proteinuria in IgA Nephropathy. Kidney Int. Rep..

[74] Mathur M., Barratt J., Chacko B., Chan T.M., Kooienga L., Oh K.-H., Sahay M., Suzuki Y., Wong M.G., Yarbrough J. (2024). A Phase 2 Trial of Sibeprenlimab in Patients with IgA Nephropathy. N. Engl. J. Med..

[75] Merrill J.T., Shanahan W.R., Scheinberg M., Kalunian K.C., Wofsy D., Martin R.S. (2018). Phase III trial results with blisibimod, a selective inhibitor of B-cell activating factor, in subjects with systemic lupus erythematosus (SLE): Results from a randomised, double-blind, placebo-controlled trial. Ann. Rheum. Dis..

[76] Barratt J., Hislop C., Pennington J. (2016). FR-PO1128 effects of blisibimod, a selective inhibitor of B-cell activating factor, in patients with IgA nephropathy. J. Am. Soc. Nephrol..

[77] Kaegi C., Steiner U.C., Wuest B., Crowley C., Boyman O. (2020). Systematic Review of Safety and Efficacy of Atacicept in Treating Immune-Mediated Disorders. Front. Immunol..

[78] Barratt J., Tumlin J., Suzuki Y., Kao A., Aydemir A., Pudota K., Jin H., Gühring H., Appel G. (2022). Randomized Phase II JANUS Study of Atacicept in Patients With IgA Nephropathy and Persistent Proteinuria. Kidney Int. Rep..

[79] Lafayette R., Barbour S., Israni R., Wei X., Eren N., Floege J., Jha V., Kim S.G., Maes B., Phoon R.K. (2024). A phase 2b, randomized, double-blind, placebo-controlled, clinical trial of atacicept for treatment of IgA nephropathy. Kidney Int..

[80] Dhillon S. (2021). Telitacicept: First Approval. Drugs.

[81] Yao X., Ren Y., Zhao Q., Chen X., Jiang J., Liu D., Hu P. (2021). Pharmacokinetics analysis based on target-mediated drug distribution for RC18, a novel BLyS/APRIL fusion protein to treat systemic lupus erythematosus and rheumatoid arthritis. Eur. J. Pharm. Sci..

[82] Lv J., Liu L., Hao C., Li G., Fu P., Xing G., Zheng H., Chen N., Wang C., Luo P. (2022). Randomized Phase 2 Trial of Telitacicept in Patients With IgA Nephropathy With Persistent Proteinuria. Kidney Int. Rep..

[83] Zan J., Liu L., Li G., Zheng H., Chen N., Wang C., Xie D., Zuo L., Li R., Zhang P. (2024). Effect of telitacicept on circulating Gd-IgA1 and IgA-containing immune complexes in IgA nephropathy. Kidney Int. Rep..

[84] Madan A., Park I., Yalavarthy R., Mandayam S., Kulkarni H., Barratt J., Davies R., Enstrom A., Thomas H., Li J. (2024). #1342 Updated results from the RUBY-3 study of povetacicept, an enhanced dual BAFF/APRIL antagonist, in autoantibody-associated glomerulonephritis. Nephrol. Dial. Transplant..

[85] Barratt J., Kooienga L., Hour B., Agha I., Schwartz B., Sorensen B., Lo J., King A., Sathaliya T., Iyer S. (2022). MO212: Updated interim results of a phase 1/2 study to investigate the safety, tolerability, pharmacokinetics, pharmacodynamics and clinical activity of BION-1301 in patients with IgA nephropathy. Nephrol. Dial. Transplant..

[86] Barratt J., Kim S.G., Agha I., Kooienga L., Madan A., Ruiz-Ramon P., Thomas H., Workeneh B., Narayanan R., Sorensen B. (2023). WCN23-1175 updated interim results of a phase 1/2 study of BION-1301 in patients with IgA nephropathy. Kidney Int. Rep..

[87] Kim S.G., Lee E.Y., Narayanan R., Sorensen B., Schwartz B.M., King A.J., Jones-Burton C., Barratt J. (2023). WCN23-1107 a phase 1/2 multicenter study to investigate the safety, tolerability, pharmacokinetics and pharmacodynamis of BION-1301 in healthy volunteers and adults with iga nephropathy. Kidney Int. Rep..

[88] Carvalho-Santos A., Kuhnert L.R.B., Hahne M., Vasconcellos R., Carvalho-Pinto C.E., Villa-Verde D.M.S. (2024). Anti-inflammatory role of APRIL by modulating regulatory B cells in antigen-induced arthritis. PLoS ONE.

[89] Thompson N., Isenberg D.A., Jury E.C., Ciurtin C. (2016). Exploring BAFF: Its expression, receptors and contribution to the immunopathogenesis of Sjögren’s syndrome. Rheumatology.

